# Remarkably Enhanced Lattice Oxygen Participation in Perovskites to Boost Oxygen Evolution Reaction

**DOI:** 10.3390/nano13050905

**Published:** 2023-02-27

**Authors:** Aditya Narayan Singh, Amir Hajibabaei, Muhammad Hanif Diorizky, Qiankai Ba, Kyung-Wan Nam

**Affiliations:** 1 Department of Energy and Materials Engineering, Dongguk University, Seoul 04620, Republic of Korea; 2 Yusuf Hamied Department of Chemistry, University of Cambridge, Lensfield Road, Cambridge CB2 1EW, UK; 3 Center for Superfunctional Materials, Department of Chemistry, Ulsan National Institute of Science and Technology (UNIST), 50, UNIST-gil, Ulsan 44919, Republic of Korea; 4 Center for Next Generation Energy and Electronic Materials, Dongguk University, Seoul 04620, Republic of Korea

**Keywords:** perovskites, oxygen evolution reaction, lattice oxygen mechanism, adsorbate evolution mechanism

## Abstract

Enhancing the participation of the lattice oxygen mechanism (LOM) in several perovskites to significantly boost the oxygen evolution reaction (OER) is daunting. With the rapid decline in fossil fuels, energy research is turning toward water splitting to produce usable hydrogen by significantly reducing overpotential for other half-cells’ OER. Recent studies have shown that in addition to the conventional adsorbate evolution mechanism (AEM), participation of LOM can overcome their prevalent scaling relationship limitations. Here, we report the acid treatment strategy and bypass the cation/anion doping strategy to significantly enhance LOM participation. Our perovskite demonstrated a current density of 10 mA cm^−2^ at an overpotential of 380 mV and a low Tafel slope (65 mV dec^−1^) much lower than IrO_2_ (73 mV dec^−1^). We propose that the presence of nitric acid-induced defects regulates the electronic structure and thereby lowers oxygen binding energy, allowing enhanced LOM participation to boost OER significantly.

## 1. Introduction

In the societal pursuit of achieving a clean, green, and carbon-neutral society, the electrolysis of water ($2H_{2}O=2H_{2}+O_{2}$) producing useful hydrogen is envisioned as playing an important role, as this does not generate harmful by-products. However, oxygen is often considered a by-product of an anodic half-cell reaction during water electrolysis. Of the two reactions of water oxidation, namely hydrogen and oxygen evolution reaction (HER and OER), the OER with the 4-electron process is often sluggish and challenging as it involves the coupling of multiple electron–proton transfers and the formation of two oxygen−oxygen bonds, [1,2] and thus determines the overall rate kinetics of water. Ir- and Ru-based compounds [3,4,5,6,7] are the necessary materials among the best electrocatalysts for OER applications; however, their commercial viability is restricted due to scarcity and prohibitive cost. These concerns have driven the exploration of several low-cost transition metal-based materials, among which transition metal (TM) oxides featuring a perovskite structure have attracted greater attention due to excellent OER performances, especially in alkaline media [8]. Recent reports have suggested that fine-tuning the perovskites can even surpass the best Ir/Ru-based OER catalysts [9,10,11,12].

Perovskites with an ABO_3_ type structure (A is alkaline/rare earth ion and B is any TM) have gained significant attention due to their high catalytic activities compared to precious metal-based catalysts such as RuO_2_ and IrO_2_ [13,14]. Additionally, apart from the conventional AEM, where the oxygen intermediates binding to the reactive surfaces should be neither too strong nor too weak to achieve optimal performances according to Sabatier’s principle [15]; the filling of the 3d electron having an e_g_ symmetry of surface TM cations has also been proposed. Following this, several electrocatalysts such as Ba_0.5_Sr_0.5_Co_0.8_Fe_0.2_O_3-δ_ (BSCF) [16], SrFeO_3_, SrNb_0.1_Co_0.7_Fe_0.2_O_3-δ_ (SNCF) [17], CaFeO_3_, and CaMnO_2.5_ [18] have been explored. However, theoretical calculations have tended to end with the perovskite being completely dominated by AEM due to the larger overpotential (η) for OER because of the scaling relationship between oxygen intermediates [19]. Though cation doping and surface engineering have reported some improvements, it still has a long way to go [20,21].

Recently, a novel mechanism called LOM has been proposed, which is similar to anionic redox in batteries [22]. This mechanism involves the direct participation of active intermediate oxygen anions from the lattice and has been supported by ^18^O isotope and theoretical calculations [23,24,25]. Notably, it is expected that the electrocatalyst exhibiting LOM can bypass the inherent limitation imposed by AEM, thereby significantly improving OER performances. Thus, there is a pressing need to develop catalysts following the LOM pathway.

**Scheme 1:**
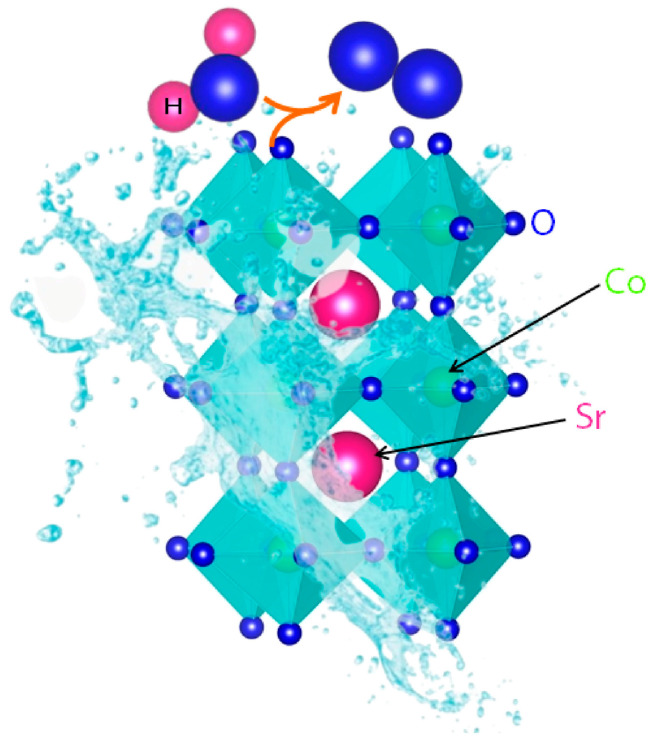
Schematic illustration of facile O_2_ desorption from the surface.

Herein, we choose a well-known strontium cobaltite, i.e., SrCoO_3-δ_ (SCO), an LOM-dominated perovskite that has been recently studied experimentally and theoretically for its high OER activity [10,25,26,27]. Previous studies have shown that doping with various cations, such as Nb, Fe, Ba, Ca, and Zr [17,28,29,30], can enhance LOM participation in SCO for OER, with Si being the most effective [10]. Here, we adopt a simple acid (HNO_3_) treatment strategy to introduce defects to significantly increase the OER performance of SCO much higher than the reported perovskites electrocatalysts (Table S1). Controlled acid treatment is an established technique to introduce defects in multi-walled carbon nanotubes [31], graphene [32], MOF [33], semiconductors [34], and various other fields to significantly improve their energy applications. Our acid-treated SCO (SCO-A) shows enhanced OER performances compared to SCO, due to its defects, and promotes the facile desorption of O_2_ from the surface (Scheme 1).

## 2. Materials and Methods

### 2.1. Materials and Reagents

Strontium nitrate (10042-76-9) and Cobalt nitrate hexahydrate (10026-22-9) were purchased from Sigma-Aldrich (St. Louis, MO, USA) and Junsei (Ohmano-Cho, Koshigaya, Saitama, Japan), respectively. Acetone was purchased from Samchun Chemicals (Gangnam-gu, Seoul, Republic of Korea). The molar mass of Sr: Co was taken as 2.1163 and 2.9103 gm, respectively.

### 2.2. Syntheses of OER Active Perovskite Materials

Freshly and extra pure-grade precursor salts were taken and ground manually for 5–10 minutes to obtain a fine powder. Later, they were ball-milled with acetone for ten hours using a two-place programmable ball mill (Daihan Scientific) at a rotation of 200 rpm. Subsequently, the powder samples were dried at 60 °C. The obtained powders were calcined in a tube furnace at 1200 °C for 10 hours at 5 °C/min in the ambient atmosphere. Finally, the furnace was cooled down to room temperature, and samples were manually ground to obtain a fine powder of SCO. SCO-A was obtained by the HNO_3_ (0.01 M) treatment of SCO powders with 20 μL in 15 mL DI water with constant stirring at 60 °C in a Petri dish. The sample was then dried and manually ground to obtain the SCO-A.

### 2.3. Characterization

High-power powder X-ray diffraction (HP-PXRD) data were collected to identify the crystal structures in the 2θ range of 10–80°, using a Rigaku X-ray diffractometer (3 phase, 380 V, 18 kW) attached with Cu Kα radiation (λ = 1.54 Å). To obtain XRD data, powder samples were deposited on a glass substrate. Scanning electron microscope (SEM) was taken on SU8220 Cold FE-SEM (Hitachi) instrument. A transmission electron microscope (TEM) was used to obtain morphological shapes and sizes. Samples for TEM analysis were prepared by a conventional method of ultra-sonication of powdered samples dispersed in ethanol. The finely dispersed samples were then drop-cast on a TEM Cu-grid.

### 2.4. Electrochemical Characterization: OER

The characterization of the electrochemical data was performed on a three-electrode setup with a system using a VSP (BioLogic Science Instruments, Inc.). The calibrated Hg/HgO (Figure S1 (Supplementary Materials)) and a graphitic rod were used as reference and counter electrodes, respectively. The chosen working electrode in our study was the glassy carbon electrode GCE (0.196 cm^2^). 0.285 mg cm^−2^ sample loading was obtained for the working electrode by the drop casting method. The entire set was kept in an oxygen environment for 20 minutes to ensure that the experiment was conducted under equilibrium H_2_O/O_2_. Cyclic voltammogram (CV) cycles were carried out to stabilize the electrode for further data collection. The linear sweep voltammograms’ data were obtained at a scan rate of 2 mV s^−1^. The interconversion voltage calculations for reversible hydrogen electrode data were obtained by Equation (1):

(1) $$E_{RHE}=E_{\mathrm{Hg}/\mathrm{HgO}}+E_{\mathrm{Hg}/\mathrm{HgO}}^{0}+0.059\times \mathrm{pH}$$

To obtain Tafel slopes and plots, the following Equation (2) was used:

η = b log j + C(2)

where η, b, j, and C represent overpotential, Tafel slope, current density, and intercept, respectively.

The overpotential (η) was calculated using Equation (3):

η = E (vs. R.H.E) – 1.23.(3)

## 3. Results and Discussions

**Figure 1:**
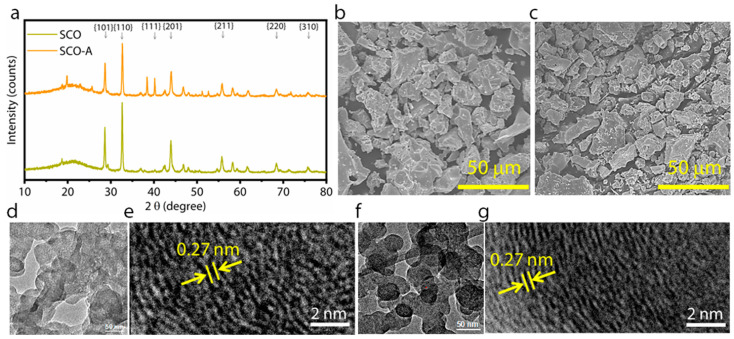
Crystal structure and physical characterizations. (**a**) X-ray diffraction pattern of perovskite SCO and SCO-A. SEM images of (**b**) SCO; (**c**) SCO-A. TEM images of (**d**–**e**) SCO; (**f**–**g**) SCO-A.

To comparatively study SCO and SCO-A, we synthesized them by two-stage ball-mill-assisted solid-state reaction (Section 2). The crystal structure (Figure 1a) of both the perovskite materials has a cubic-phase structure, and characteristic peaks can be indexed to the Pm-3m space group with slight impurity phases of Co_3_O_4_, almost in line with the previously reported literature [35]. The corresponding featured peaks at 28.56°, 32.78°, 40.18°, 43.94°, 55.84°, 68.4°, and 75.64° are designated to (101), (110), (111), (201), (211), (220), and (310) planes and can be indexed with JCPDS card no. 48-0857, which is consistent with the previous report [36]. The slight decline in peak intensity in SCO-A, compared to SCO, indicates a reduced crystallinity, possibly due to defects arising from acid treatment. Decreased peak intensity due to defects/disorders has also been reported in energy materials [37]. SEM images of both the perovskites show a similar morphology with average particle size down to ~20–30 micrometers (Figure 1b,c). However, there are more exposed defective surfaces/areas in SCO-A (Figure S2). The nanoscale defective zone significantly improves the electrochemical performances in many energy materials by offering larger active surface areas for electrochemical reactions [38]. Additionally, in SCO-A, some fine particles can be seen on the larger sized particles, making them slightly rough, probably due to the implantation of defects on the surface. A controlled amount of defective surfaces usually assists in better OER performances (discussed later) [39]. Their TEM images (Figure 1d–g) show no noticeable differences in their overall morphology. The lattice fringe distance of 0.27 nm corresponds to characteristic (110) planes obtained from XRD data.

**Figure 2:**
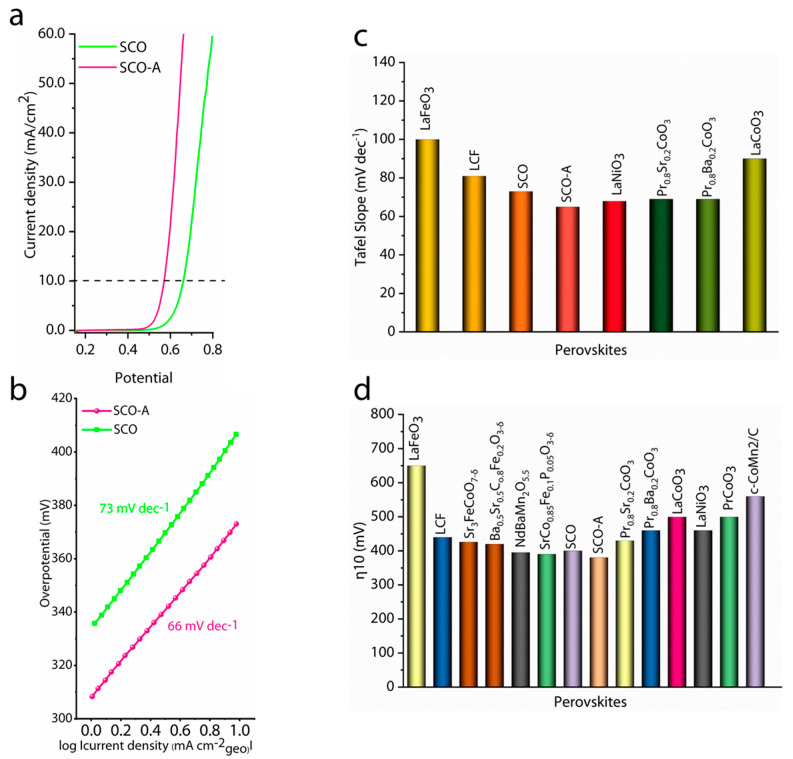
Electrocatalytic performances. (**a**) LSV polarization curve to demonstrate OER kinetic currents for SCO and SCO-A obtained in an O_2_-saturated 1M KOH environment. (**b**) Tafel plots. (**c**) A comparison of the Tafel plots for various electrodes. (**d**) Overpotential for different cathodes at the standard current density of 10 mA cm^−2^.

Inspired by the implantation of surface defects reflected in rough particle surfaces as observed in SEM, we carried out its electrochemical performances. The OER data were obtained on a three-electrode setup (Section 2). Notably, the LSV curve of SCO-A shows an increased current density as compared to SCO, consuming a low η of 380 mV compared to 400 mV for SCO to reach a benchmark current density of 10 mA cm^−2^ (Figure 2a and Figure S3). This value becomes more rifted as the current density continues to increase. For instance, to achieve a current density of 60 mA cm^−2^, while SCO-A requires 663 mV, SCO consumes 796 mV, almost 133 mV in excess, to reach the same current density. Strikingly, the η necessary to achieve the set benchmark current density for SCO-A is not only lesser than several dopped perovskites such as Si-SCO, SrCo_0.85_Fe_0.1_P_0.05_O_3-δ_, and LaCo_0.2_Fe_0.8_O_3_, but is comparable to noble metal catalyst (Table S1). To have a deeper insight into the electrochemical performances, we obtained the Tafel slopes of the perovskites. The SCO-A has a lower slope (65 mV dec^−1^) than SCO (73 mV dec^−1^) and several other perovskites engaged in OER applications (Figure 2b,c). Interestingly, this value is much lower than the benchmark IrO_2_ (73 mV dec^−1^) [40]. The decline in the Tafel slope is possibly due to a reduction in onset potential in SCO-A (shown later). From the OER electrochemical results discussed so far, SCO-A stands out among several reported perovskites utilized as an OER electrocatalyst (Figure 2d).

**Figure 3:**
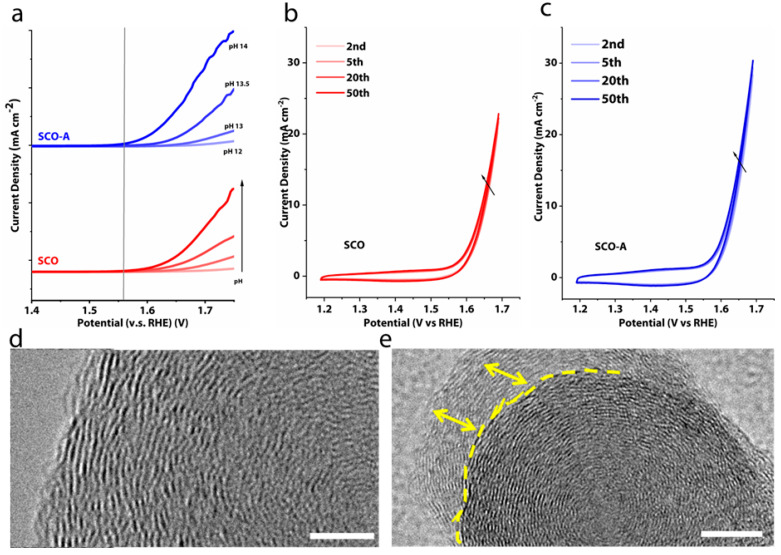
The oxygen evolution mechanism studies for SCO and SCO-A. (**a**) OER kinetics of SCO and SCO-A in O_2_-saturated 1M KOH environment with varying pH. CV curves of (**b**) SCO. (**c**) SCO-A. TEM images after 50^th^ cycles: (**d**) SCO. (**e**) SCO-A (scale bar 2 nm).

pH dependency is another aspect that confirms the presence of LOM participation in perovskites and other electrocatalysts [35]. We compare the onset potentials of our perovskites in an O_2_-saturated KOH environment, with pH varying from 12 to 14 (Figure 3a). It is interesting to note that the onset potential of SCO-A slightly improves with a gradual increase in OER activity common to both perovskites with the increasing pH, indicative of the pH dependence of the OER kinetics and hence LOM participation. The improvement in the onset potential indicates that the presence of surface defects can modify the electronic structures and can significantly assist in promoting reaction kinetics. Such a shift in onset potential due to the presence of defects has recently been reported for hydrogen evolution reaction (HER) [41].

Further evidence supporting improved LOM participation can come from the CV cycling during the initial 50 cycles (Figure 3b,c, Figure S4, and Supplementary discussion S1). The surface dynamic reconstruction of the catalytic surface area is another fingerprint of LOM in electrocatalysts widely reported in perovskites that significantly boosts their OER performances [21,42]. The area enclosed in CV curves represents a pseudocapacitive feature that directly dictates the available surface area for the reactions. The pseudocapacitive and OER current density continuously increase for the perovskites during the continuous cycling, indicating surface amorphization. Such a phenomenon has also been recently reported in other perovskites [43]. Notably, this change is more significant in SCO-A, indicating a higher tendency of LOM participation in OER. To support the surface amorphization, TEM images were taken after the 50th cycle (Figure 3d,e). The surface amorphization in SCO-A is significantly higher (≈3 nm) than in SCO and its pristine state. These results further support the involvement of lattice oxygen in acid-treated SCO, though at the expense of surface instability.

**Figure 4:**
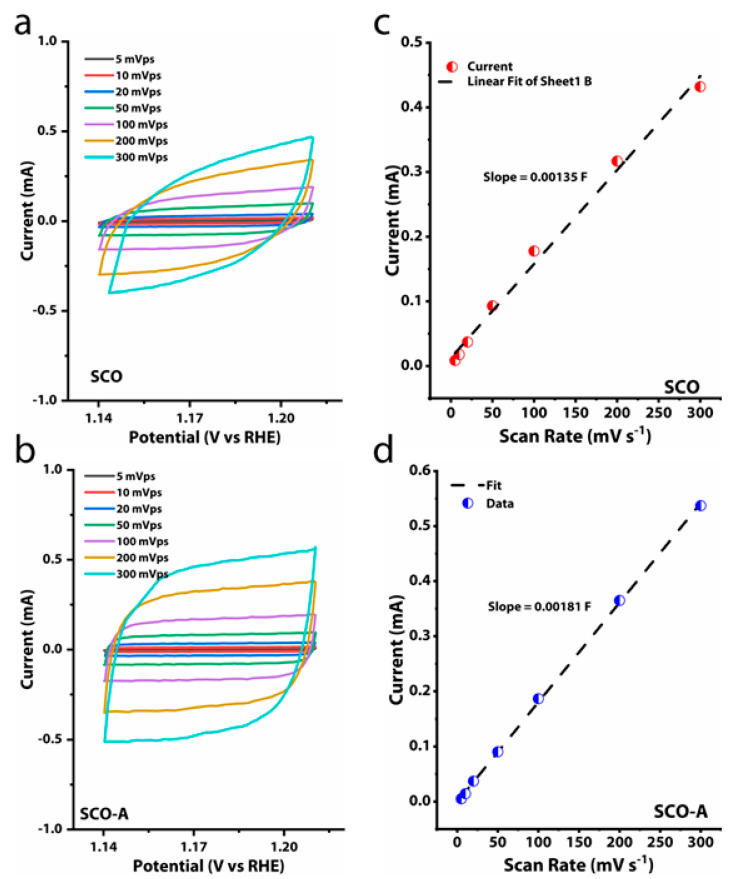
CV curves and the Tafel Slope. CV curves at different scan rates for (**a**) SCO; (**b**) SCO-A. Tafel plots for (**c**) SCO. (**d**) SCO-A.

To further support the enhanced participation of LOM in SCO-A, we conducted CV cycling at various scan rates (Figure 4a,b). From the received wisdom, it is now well established that the area enclosed within the CV curve ensembles the available active surface area and is a strong tool to determine the activity of a given catalyst [44]. It is obvious from the CV curves that the active electrochemical surface area in SCO-A is significantly larger than at a given scan rate for SCO. To further corroborate this idea, the double-layer capacitance (C_dl_) was obtained (Figure 4c,d). The double-layer capacitance of SCO-A (0.00181 F) is ~1.3 times higher than SCO (0.00135 F), demonstrating a high density of active sites for the former, which can further boost the OER activities.

Accelerated OER kinetics associated with oxygen diffusion in SCO-A are related to defects generated by the facile desorption of oxygen from the surface and dominant LOM. Our previous studies have shown that the easy diffusion of oxygen from the surface can be triggered by either cation doping or implanting defects in the crystal lattice [4,7]. In this study, we intentionally created surface defects in the perovskite through acid treatment (Section 2) under controlled conditions sufficient not to destroy the perovskite lattice. Although predicting the precise mechanism is complicated, we suggest that in the presence of nitric acid and the impregnation of defects, the dielectric constant of the environment changes, regulating the electronic structure and lowering the binding energy of oxygen. We support this idea as the presence of defects is well known to reduce the free-energy barrier, thus augmenting the OER kinetics [45].

## 4. Summary and Conclusions

To summarize, the authors employed a simple strategy for synthesizing perovskites through a solid-state route and used acid treatment to create defects, which enhanced the lattice oxygen mechanism and improved the oxygen evolution reaction. By opting for a simple strategy, we bypassed the idea of precise A- or B-site doping, as in several perovskites, which requires fine adjustments in precursors and characterizations to confirm the actual doping site. Our acid-treated perovskite (SCO-A) performed much better than the various doped perovskites in the same environment on the GCE electrode, requiring just 380 mV to achieve benchmark current density (10 mA cm^−2^), and exhibited a Tafel slope (65 mV dec^−1^) much lower than noble metal IrO_2_ (73 mV dec^−1^). The enhanced performances of our acid-treated perovskite emanate from the following features. First, the implantation of defects and morphological changes, such as rough surfaces, assists in better OER kinetics. Secondly, the induction of defects shifted the onset potential much earlier than in SCO, further supporting an enhanced OER. Thirdly, the surface amorphization creates stresses that can be accommodated in our SCO-A to a certain extent, owing to the presence of defects. In addition, we propose that in the presence of nitric acid, the dielectric constant of the material/environment changes, tuning the electronic state and possibly promoting OER. We are in the process of understanding this from the machine learning perspective and confirming our proposition. We believe that our simple strategy to create defects in the perovskites may complement the existing cation/anion doping strategies in perovskites to design novel materials for energy applications.

## Data Availability

All the experimental data presented within this article along with the supplementary information will be made available from the authors upon reasonable request.

## Supplementary Materials

The following supporting information can be downloaded at: https://www.mdpi.com/article/10.3390/nano13050905/s1, Figure S1: Calibration of the Hg/HgO reference electrode. For all measurements, Hg/HgO was used as the reference electrode. The Hg/HgO calibration with respect to the reversible hydrogen electrode (RHE) was performed in the high-purity H_2_ saturated electrolyte with Pt wire as the working electrode. LSV was run at a rate of 1 mV s^−1^, and potential at zero mA current was taken to be the thermodynamic potential for hydrogen evolution reactions. So, in 1M KOH, E (RHE) = E(Hg/HgO) + 0.905; Figure S2: TEM image showing large area defective zone in SCO-A; Figure S3: (a) LSV polarization curve of IrO_2_. (b) Tafel plot of IrO_2_; Figure S4: LSV plot showing OER performances after 1st and 50th CV cycles; Table S1: A comparative chart for overpotential (η) of perovskite materials used as OER electrocatalyst; S1: The OER stability data. References [28,29,30,46,47,48,49,50,51,52] are cited in the Supplementary Materials.
