# Supplementary material for: Remarkably Enhanced Lattice Oxygen Participation in Perovskites to Boost Oxygen Evolution Reaction
Source: Nanomaterials (Basel). 2023 Feb 27;13(5):905. doi: 10.3390/nano13050905 (PMC10005787; doi:10.3390/nano13050905)
Supplement: Supplementary file 1 [file nanomaterials-13-00905-s001.zip › nanomaterials-2235014-supplementary.pdf]

Supplementary Information for

Remarkably Enhanced Lattice Oxygen Participation in Perovskites to Boost Oxygen Evolution Reaction

Aditya Narayan Singh [1,*], Amir Hajibabaei [2], Muhammad Hanif Diorizky [3], Qiankai Ba [3] and Kyung-Wan Nam [1,4,*]

[1] Department of Energy and Materials Engineering, Dongguk University-Seoul, Seoul 04620, Republic of Korea
[2] Yusuf Hamied Department of Chemistry, University of Cambridge, Lensfield Road, Cambridge CB2 1EW, UK
[3] Center for Superfunctional Materials, Department of Chemistry, Ulsan National Institute of Science and Technology (UNIST), 50, UNIST-gil, Ulsan 44919, Republic of Korea
[4] Center for Next Generation Energy and Electronic Materials, Dongguk University—Seoul, Seoul 04620, Republic of Korea
* Correspondence: aditya@dongguk.edu (A.N.S.); knam@dongguk.edu (K.-W.N.)

Supplementary discussion.

S1. The OER stability data

The OER stability data were recorded after 1st and 50th cycles, which shows that SCO-A is quite stable and defects are not in excessive amounts to disrupt the crystal structures rather than the surface defects to improve LOM participation in perovskite to boost OER kinetics (Figure S4).

Table S1. A comparative chart for overpotential (η) of perovskite materials used as OER electrocatalyst.

**Catalysts**	**η @ 10 mA cm^{-2} (mV)**	**Condition (Electrolyte)**	**Refs.**
SCO	400	1 M KOH	this work
SCO-A	380	1 M KOH	this work
$Ba_{0.5}Sr_{0.5}Co_{0.8}Fe_{0.2}O_{3-\delta}$ (BSCF)	420	1M KOH	[1]
CQDs@BSCF-NFs	350	1M KOH	[1]
$SrCo_{0.85}Fe_{0.1}P_{0.05}O_{3-\delta}$	390	1M KOH	[2]
$LaCo_{0.2}Fe_{0.8}O_3$ (LCF)	440	1M KOH	[3]
S-LCF	360	1M KOH	[3]
$NdBaMn_2O_{5.5}$	395	1 M KOH	[4]
$LaCoO_3$	390	1 M KOH	[5]
$La_{0.5}(Ba_{0.4}Sr_{0.4}Ca_{0.2})_{0.5}Co_{0.8}Fe_{0.2}O_{3-\delta}$/rGO	338	1 M KOH	[6]
$Sr_3FeCoO_{7-\delta}$	426	1 M KOH	[7]
NMP-treated Sr3FeCoO7-δ	343	1 M KOH	[7]
$BaCo_{0.5-x}Fe_{0.5-x}Zr_xY_xO_{3-\delta}$(BCF(ZY)$_x$, x = 0.0−0.2)	320	1 M KOH	[8]
$K(MgMnFeCoNi)F_3$	369	1 M KOH	[9]
$LaFeO_3$	650	1 M KOH	[10]
$LaNiO_3$	460	1 M KOH	[10]
$PrCoO_3$	500	1 M KOH	[10]

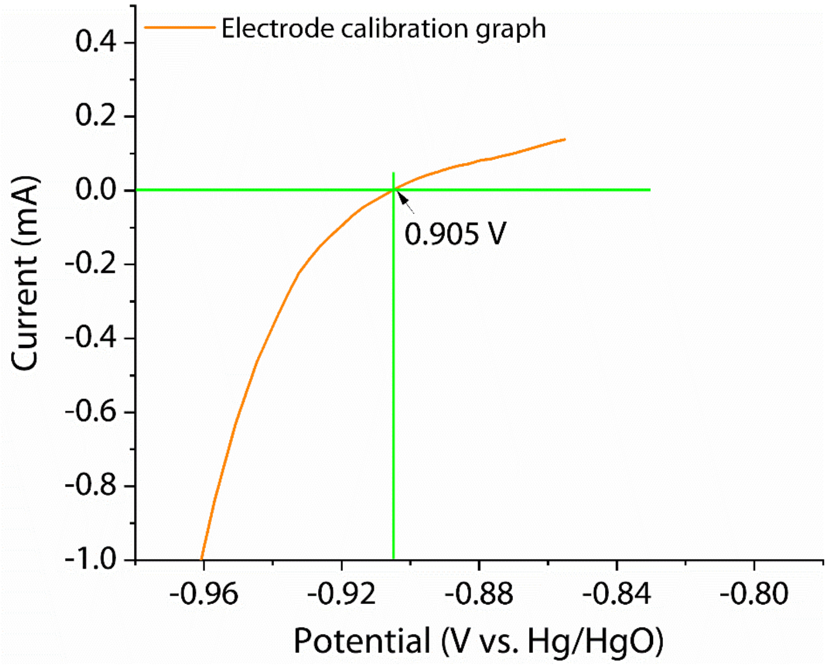

Figure S1. Calibration of the Hg/HgO reference electrode. For all measurements, Hg/HgO was used as the reference electrode. The Hg/HgO calibration with respect to the reversible hydrogen electrode (RHE) was performed in the high-purity H_2 saturated electrolyte with Pt wire as the working electrode. LSV was run at a rate of 1 mV s^{-1}, and potential at zero mA current was taken to be the thermodynamic potential for hydrogen evolution reactions. So, in 1M KOH, E (RHE) = E(Hg/HgO) + 0.905.

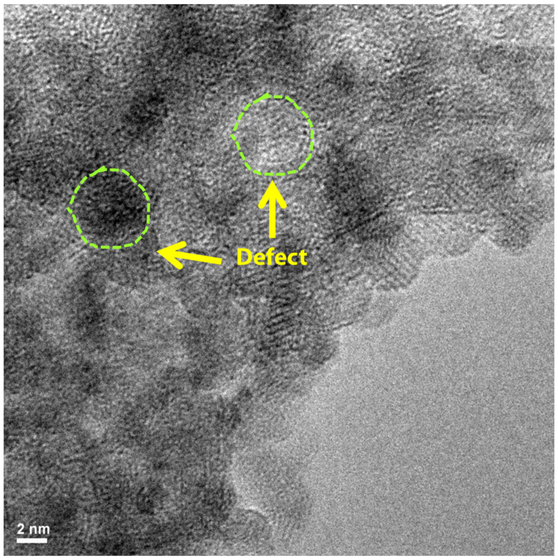

Figure S2. TEM image showing large area defective zone in SCO-A.

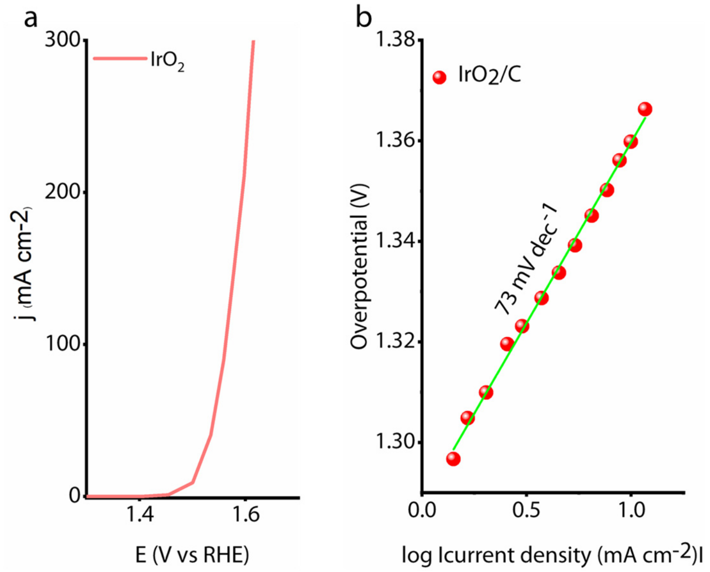

Figure S3. (a) LSV polarization curve of IrO_2. (b) Tafel plot of IrO_2.

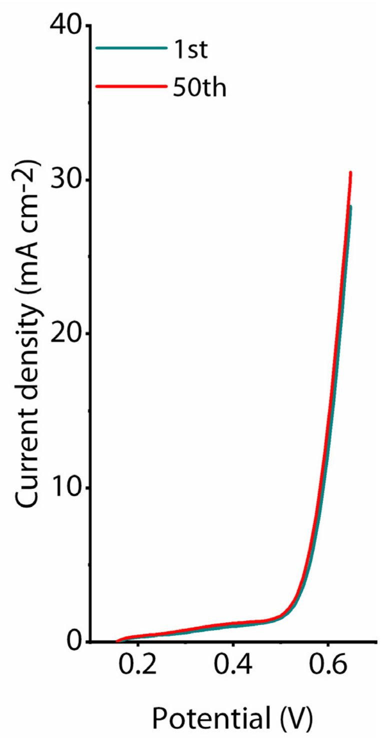

Figure S4. LSV plot showing OER performances after 1st and 50th CV cycles.

References

1. Li, G.; Hou, S.; Gui, L.; Feng, F.; Zhang, D.; He, B.; Zhao, L. Carbon quantum dots decorated Ba0.5Sr0.5Co0.8Fe0.2O3-δ perovskite nanofibers for boosting oxygen evolution reaction. *Appl. Catal. B: Environ.* **2019**, *257*, 117919, doi:https://doi.org/10.1016/j.apcatb.2019.117919.
2. Du, X.; Ai, H.; Chen, M.; Liu, D.; Chen, S.; Wang, X.; Lo, K.H.; Pan, H. PLD-fabricated perovskite oxide nanofilm as efficient electrocatalyst with highly enhanced water oxidation performance. *Appl. Catal. B: Environ.* **2020**, *272*, 119046, doi:https://doi.org/10.1016/j.apcatb.2020.119046.
3. Tang, L.; Chen, Z.; Zuo, F.; Hua, B.; Zhou, H.; Li, M.; Li, J.; Sun, Y. Enhancing perovskite electrocatalysis through synergistic functionalization of B-site cation for efficient water splitting. *Chem. Eng. J.* **2020**, *401*, 126082, doi:https://doi.org/10.1016/j.cej.2020.126082.
4. Wang, J.; Gao, Y.; Chen, D.; Liu, J.; Zhang, Z.; Shao, Z.; Ciucci, F. Water Splitting with an Enhanced Bifunctional Double Perovskite. *ACS Catal.* **2018**, *8*, 364-371, doi:10.1021/acscatal.7b02650.
5. Zhao, T.; Wang, Y.; Chen, X.; Li, Y.; Su, Z.; Zhao, C. Vertical Growth of Porous Perovskite Nanoarrays on Nickel Foam for Efficient Oxygen Evolution Reaction. *ACS Sustain. Chem. Eng.* **2020**, *8*, 4863-4870, doi:10.1021/acssuschemeng.0c00060.
6. Hua, B.; Li, M.; Zhang, Y.-Q.; Sun, Y.-F.; Luo, J.-L. All-In-One Perovskite Catalyst: Smart Controls of Architecture and Composition toward Enhanced Oxygen/Hydrogen Evolution Reactions. *Adv. Energy Mater.* **2017**, *7*, 1700666, doi:https://doi.org/10.1002/aenm.201700666.
7. Xu, K.; Song, F.; Gu, J.; Xu, X.; Liu, Z.; Hu, X. Solvent-induced surface hydroxylation of a layered perovskite Sr3FeCoO7−δ for enhanced oxygen evolution catalysis. *J. Mater. Chem. A* **2018**, *6*, 14240-14245, doi:10.1039/C8TA04976A.
8. Li, X.; Zhang, J.; Feng, Q.; Pu, C.; Zhang, L.; Hu, M.; Zhou, X.; Zhong, X.; Yi, W.; Tang, J.; et al. Redox inactive ion meliorated BaCo0.4Fe0.4Zr0.1Y0.1O3−δ perovskite oxides as efficient electrocatalysts for the oxygen evolution reaction. *J. Mater. Chem. A* **2018**, *6*, 17288-17296, doi:10.1039/C8TA05599H.
9. Wang, T.; Chen, H.; Yang, Z.; Liang, J.; Dai, S. High-Entropy Perovskite Fluorides: A New Platform for Oxygen Evolution Catalysis. *J. Am. Chem. Soc.* **2020**, *142*, 4550-4554, doi:10.1021/jacs.9b12377.
10. Matienzo, D.D.; Kutlusoy, T.; Divanis, S.; Di Bari, C.; Instuli, E. Benchmarking Perovskite Electrocatalysts' OER Activity as Candidate Materials for Industrial Alkaline Water Electrolysis. *Catalysts* **2020**, *10*, 1387.
